# The spatial scale of genetic subdivision in populations of *Ifremeria nautilei*, a hydrothermal-vent gastropod from the southwest Pacific

**DOI:** 10.1186/1471-2148-11-372

**Published:** 2011-12-22

**Authors:** Andrew D Thaler, Kevin Zelnio, William Saleu, Thomas F Schultz, Jens Carlsson, Clifford Cunningham, Robert C Vrijenhoek, Cindy L Van Dover

**Affiliations:** 1 Marine Laboratory, Nicholas School of the Environment, Duke University, 135 Duke Marine Lab Rd., Beaufort, NC 28516, USA; 2 School of Biological, Earth and Environmental Sciences Distillery Fields, North Mall University College Cork, Ireland; 3 Department of Biology, Duke University, Box 90338, Durham, NC 27708, USA; 4 Monterey Bay Aquarium Research Institute, 7700 Sandholdt Road, Moss Landing, CA 95039, USA

## Abstract

**Background:**

Deep-sea hydrothermal vents provide patchy, ephemeral habitats for specialized communities of animals that depend on chemoautotrophic primary production. Unlike eastern Pacific hydrothermal vents, where population structure has been studied at large (thousands of kilometres) and small (hundreds of meters) spatial scales, population structure of western Pacific vents has received limited attention. This study addresses the scale at which genetic differentiation occurs among populations of a western Pacific vent-restricted gastropod, *Ifremeria nautilei*.

**Results:**

We used mitochondrial and DNA microsatellite markers to infer patterns of gene flow and population subdivision. A nested sampling strategy was employed to compare genetic diversity in discrete patches of *Ifremeria nautilei *separated by a few meters within a single vent field to distances as great as several thousand kilometres between back-arc basins that encompass the known range of the species. No genetic subdivisions were detected among patches, mounds, or sites within Manus Basin. Although *I. nautilei *from Lau and North Fiji Basins (~1000 km apart) also exhibited no evidence for genetic subdivision, these populations were genetically distinct from the Manus Basin population.

**Conclusions:**

An unknown process that restricts contemporary gene flow isolates the Manus Basin population of *Ifremeria nautilei *from widespread populations that occupy the North Fiji and Lau Basins. A robust understanding of the genetic structure of hydrothermal vent populations at multiple spatial scales defines natural conservation units and can help minimize loss of genetic diversity in situations where human activities are proposed and managed.

## Background

The spatial scales at which individuals within a population interact and the geographic extent of larval dispersal shape the dynamics of marine populations. Dispersal capabilities of some species extend across entire ocean basins [1], but larval propagules of many other species are retained close to their source [2]. Larval development can impose limits on dispersal. Species that brood their offspring (direct development) tend to have more restricted distributions than species with long-lived, planktonic larvae [3], though exceptions exist [4]. Species that aggregate in small patches may interact and reproduce with other individuals in an area encompassing a meter or less [5] and larvae that lack broad dispersal potential may recruit to their natal population [6]. A sampling scheme that fails to account for the localized effects of self-recruiting patches may create an appearance of panmixia, even if substructure exists among patches [7].

Species dependent on deep-sea hydrothermal vents are restricted to patchy, ephemeral habitats that limit the areal extent and occurrence of populations. Hydrothermal vent fields are found on mid-ocean ridges, back-arc spreading centres, and submarine volcanoes [8]. Organisms that thrive at vents are supported by chemoautotrophic microbes that metabolize reduced compounds in the vent effluent [9]. Vent habitats are transient, at temporal scales ranging from days to hundreds of years [10], and constituent species may be subject to frequent local extinction and recolonization events [11,12]. Survival of vent species therefore depends on fast growth, rapid reproduction, and dispersal abilities that shape the diversity and genetic structure of populations [13,14].

At mid-ocean ridges, deep-sea hydrothermal vents are distributed along roughly linear axes that may function as dispersal corridors [15-17]. Geographic populations of hydrothermal vent-dependent species can be panmictic across the extent of their range (e.g., the shrimp, *Rimicaris exoculata *on the Mid-Atlantic Ridge [18-20]) but this is not always the case.

Evidence for isolation-by-distance in vent species has sometimes been ambiguous due to small sample sizes and inconsistency in the resolution of various genetic markers [21]. Considerable evidence exists for geographic subdivision associated with geomorphological features that affect different taxa to varying degrees. For example, the Easter Microplate is associated with isolation of northern and southern East Pacific Rise populations of mussels, but not of polychaete annelids [22,23]. A 2000-m long "habitat gap" across the Equator is implicated in the isolation of some East Pacific Rise species and variable impedance of gene flow in other species [23,24]. Similarly, a 350-km long ridge offset, the Blanco Transform Fault, isolates Juan de Fuca and Gorda ridge limpet populations [25]. The same barrier interacts with current regimes and is correlated with southward unidirectional gene flow in the vent polychaete *Ridgeia piscesae *[17]. Life history and behavioral attributes of various taxa result in these differing responses to shared dispersal barriers [14].

Identification of population structure at various spatial scales depends in part on the choice of genetic markers. For example, amplified fragment length polymorphisms were used to test for fine-scale differentiation among discrete patches of the tubeworm, *Riftia pachyptila*, separated by as little as 400 m in a venting area along the East Pacific Rise, although sample sizes were small (*n *< 15 per site [26]). More conservative mitochondrial and nuclear DNA sequences in *R. pachyptila *revealed panmixia at local scales and isolation-by-distance [27] at greater geographical scales [28,29].

In contrast to mid-ocean ridge systems, limited attention has been afforded to the population structure of vent organisms from western Pacific back-arc basins. These basins are distributed in a non-linear pattern, reflecting the complex tectonic history of the region [30]. Hydrothermal vents in western Pacific back-arc basins are geographically isolated from vents on the East Pacific Rise [31]. Regional isolation of species was detected among western pacific vents [30]: the Okinawa trough and Izu-Ogasawara Arc have a faunal assemblage distinct from that of other western Pacific hydrothermal vents, and the faunal composition of the Marianna Trough is distinct from that of neighbouring basins [30]. Vent species tend to be shared among Manus, North Fiji, and Lau Basin, but are distinct from species that occur at the Okinawa and Marianna Troughs or the Izu-Ogasawara Arc [30].

Because back-arc basin hydrothermal systems in the western Pacific are located on isolated ridge segments (in contrast to the linear, semi-continuous series of segments on mid-ocean ridges), it has been hypothesized that reduced connectivity among western Pacific back-arc basins may yield more endemic vent fauna within discrete back-arc basins [32]. Some species endemic to these basins appear to be panmictic across multiple basins (e.g., the mussel *Bathymodiolus brevior *[33]), whereas others are restricted to single basins (e.g., neoverrucid barnacles [34]). Provannid snail species in the genus *Alviniconcha *represent a cryptic species complex composed of at least three evolutionary lineages, one that occurs at hydrothermal vents in North Fiji Basin, one that is restricted to vents in the Marianna Trough, and one that co-occurs in both Manus and North Fiji Basin [35]. A similar pattern of strong genetic differentiation may exist within other species. To date, comprehensive efforts have not been made to characterize population structure within vent taxa of western Pacific back-arc basins.

*Ifremeria nautilei *is a provannid gastropod that occurs in Manus, North Fiji, and Lau Basins and depends on sulphur-oxidizing bacterial endosymbionts for nutrition. Sessile adults live in discrete patches near the effluent of diffuse-flow hydrothermal vents [36,30]. Females possess a specialized brood-pouch in their foot and they release ciliated pre-veliger larvae (Warén's larvae) that are hypothesized to have long-distance dispersal capabilities [37]. Preliminary studies indicated that *I. nautilei *exhibits distinct mitochondrial haplotypes in Manus and North Fiji Basins [38], but population structure has not been assessed at smaller spatial scales--among vent fields within basins (henceforth *sites*), among sulphide mounds within vent fields (henceforth *mounds*), or among discrete patches on vent mounds (henceforth *patches*).

**Figure 1:**
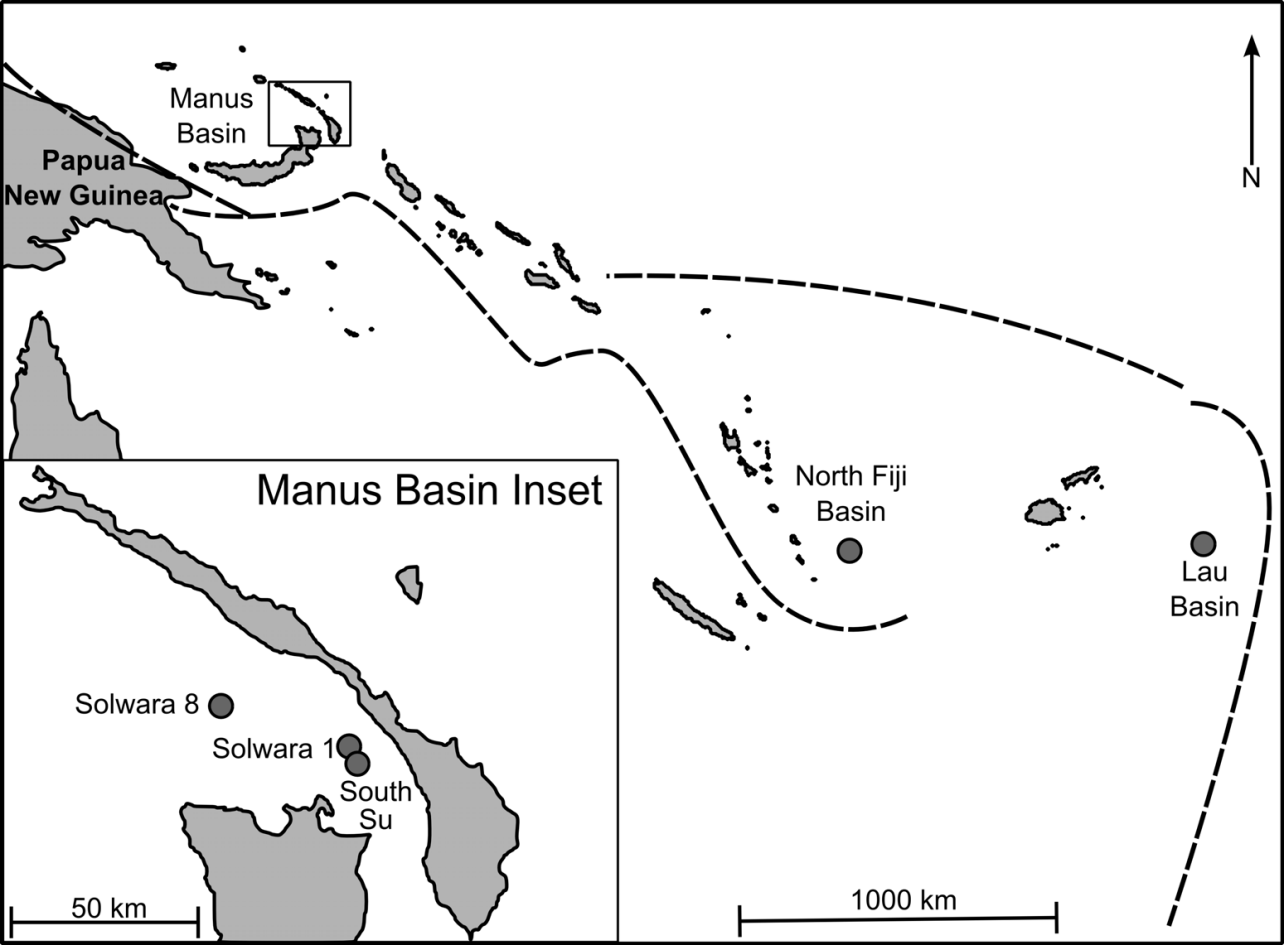
***Ifremeria nautilei *sampling locations in the western Pacific**. Grey circles are approximate location of sampling. Dashed lines represent subduction zones. Three sites sampled in Manus Basin are shown in inset.

We examined genetic population structure of *Ifremeria nautilei *from hydrothermal vents in Manus, North Fiji, and Lau Basins at multiple scales, ranging from meters to thousands of kilometres (Figure 1). A nested sampling strategy was employed within Manus Basin to test the null hypothesis that *I. nautilei *exhibits no population structure among discrete patches at spatial scales of meters to 40 kilometres. The entire Manus Basin population was then compared to North Fiji and Lau basin samples to assess the relationship between increasing spatial scales (1000 kilometres to 3500 kilometres) and genetic differentiation. Genetic markers for differentiation at these scales included partial sequences of mitochondrial cytochrome-*c*-oxidase subunit I, and an array of nuclear DNA microsatellite loci [39]. By comparing these two types of molecular markers, we can separate evolutionary processes, revealed by *COI *sequence data and dependent on mutation rates, from ecologic processes, revealed by microsatellite allele frequencies and based on the recombination of alleles with each generation [40]. If the specialized Warén's larvae produced by *Ifremeria nautilei *are adapted for long-distance dispersal [37], population structure should be minimal over all scales. Alternatively, if *I. nautilei *disperse in a manner consistent with other sessile invertebrates with specialized habitat needs [41] genetic differentiation may occur at spatial scales less than one kilometre.

## Results

### Summary statistics

*Ifremeria nautilei *were sampled from Manus, North Fiji, and Lau Basins (275 individuals total; Figure 1, Table 1). Thirty-six partial *COI *haplotypes (404 bp) were identified from 158 *Ifremeria nautilei *sampled from Manus Basin; an additional 25 haplotypes were identified from 117 individuals from North Fiji and Lau Basins. Haplotype diversity (*H_d_*) ranged from 0.59 to 0.95 (Table 2) and nucleotide diversity (π) ranged from 0.003 to 0.007. Indices of genetic diversity and tests for selection are reported in Table 2. Fu's *F_S _*values were negative, consistent with allelic excess driven by recent population expansion (but may also be indicative of a selective sweep) [42], with two exceptions (Patch 3 and Patch 16).

**Table 1:** *Ifremeria nautilei *sampling locations from Manus, North Fiji, and Lau Basin.

Basin	Site	Mound	Latitude	Longitude	Depth (m)
Manus	Solwara 8	Active 1	3° 43.740'S	151° 40.404'E	1720
		Active 2	3° 43.824'S	151° 40.458'E	1710
		Active 3	3° 43.668'S	151° 40.872'E	1650
	Solwara 1	Active 4	3° 47.436'S	152° 5.472'E	1530
		Active 5	3° 47.370'S	152° 5.778'E	1490
		Active 6	3° 47.370'S	152° 5.616'E	1480
	South Su	Active 7	3° 48.564'S	152° 6.144'E	1300
		Active 8	3° 48.492'S	152° 6.186'E	1350
		Active 9	3° 48.432'S	152° 6.306'E	1320
North Fiji	White Lady		16° 59.950'S	173° 54.950'E	1971
	White Rhino		16° 59.950'S	173° 54.950'E	1971
	Mussel Hill		16° 59.950'S	173° 54.950'E	1971
Lau	Kilo Moana		20° 03.230'S	176° 08.010'W	2620
	Tu'i Malila		20° 59.350'S	176° 34.100W	1884

All sites from North Fiji Basin are within 100 m of each other and marked with the same coordinates.

**Table 2:** *COI *summary statistics for samples of *Ifremeria nautilei *collected from Manus, North Fiji, and Lau Basin, and divided by patches, mounds, and sites within Manus Basin.

	*N*	*H*	^***H ***^***d***	^***F ***^***s***
Manus Basin	158	36	0.83 (0.02)	**-27.301**
Solwara 8	44	16	0.80 (0.06)	**-9.719**
Mound 1	15	6	0.74 (0.09)	-1.208
Mound 2	17	9	0.83 (0.09)	**-4.337**
Mound 3	12	6	0.82 (0.10)	-1.272
Solwara 1	58	16	0.79 (0.04)	**-8.427**
Mound 4	11	8	0.95 (0.05)	**-3.723**
Patch 1	7	6	0.95 (0.10)	**-3.027**
Patch 2	4	4	1.00 (0.18)	n.d.
Mound 5	26	7	0.71 (0.07)	-1.377
Patch 3	15	4	0.66 (0.08)	0.503
Patch 4	7	6	0.95 (0.10)	-0.780
Patch 5	4	3	0.83 (0.22)	n.d.
Mound 6	21	9	0.80 (0.06)	**-3.403**
Patch 6	6	3	0.60 (0.22)	n.d.
Patch 7	3	3	1.00 (0.27)	n.d.
Patch 8	12	6	0.76 (0.12)	-1.475
South Su	56	20	0.88 (0.03)	**-4.918**
Mound 7	18	11	0.91 (0.05)	**-5.865**
Patch 9	6	5	0.90 (0.16)	n.d.
Patch 10	2	2	1.00 (0.50)	n.d.
Patch 11	2	2	1.00 (0.50)	n.d.
Patch 12	8	6	0.93 (0.08)	-2.401
Mound 8	22	12	0.91 (0.04)	**-5.473**
Patch 13	5	5	1.00 (0.13)	n.d.
Patch 14	10	7	0.93 (0.06)	**-2.906**
Patch 15	7	3	0.67 (0.16)	-2.354
Mound 9	16	8	0.80 (0.09)	-2.474
Patch 16	7	4	0.81 (0.13)	1.081
Patch 17	9	7	0.92 (0.09)	**-2.952**

North Fiji Basin	81	20	0.70 (0.05)	**-14.603**
White Lady	25	11	0.83 (0.06)	**-5.655**
White Rhino	27	9	0.65 (0.10)	**-3.321**
Mussel Hill	29	8	0.61 (0.10)	**-3.033**

Lau Basin	36	10	0.59 (0.09)	**-4.918**

*N *= number of individuals, *H *= number of haplotypes, *H_d _*= haplotype diversity (standard deviation), *F_s _*= Fu's *F_s _*with significance (*P *< 0.05) indicated in bold. *F_s _*are only reported for patches with a sample size greater than or equal to 7.

Nine microsatellite loci were amplified from 40 to 66 individuals per site within Manus Basin (Additional File 1, Table S1). Total alleles per locus ranged from 3 to 23 (mean = 6.6). In permutation tests, allelic richness (*Rs*) did not vary significantly among patches, mounds, or sites (10,000 permutations, *P *> 0.05; Additional File 1, Table S1). Three loci (*Ifr040, Ifr052*, and *Ifr078*) were monomorphic at the patch level but were polymorphic among patches.

Only eight microsatellite loci were amplified from 20 to 38 individuals per site from North Fiji and Lau Basin (Additional File 1, Table S2). One locus (*Ifr086*) failed to amplify in any North Fiji or Lau Basin samples. The total number of alleles per locus ranged from 2 to 23 (mean = 6.2). In permutation tests, allelic richness (*Rs*) did not vary significantly among sites or basins (10,000 permutations, *P *> 0.05, Additional File 1, Table S2).

### Microsatellite marker quality

Tests for HWE deviation were used to assess the quality of sampled microsatellite markers. In Manus Basin, heterozygote deficiency was detected in one locus (*Ifr086*; Additional File 1, Table S1). Heterozygote excess was detected in only one locus at the site level (Solwara 1, *Ifr040*; Additional File 1, Table S1). Significant deviation from HWE was not detected at any other patch, mound, or site from Manus Basin (Additional File 1, Table S1).

Two microsatellite loci deviated significantly from Hardy-Weinberg Equilibrium at sites within North Fiji Basin (*Ifr068 *and *Ifr103*; Additional File 1, Table S2). Four microsatellite loci were not in equilibrium at the basin level (*Ifr068*, *Ifr078*, *Ifr093*, and *Ifr103*; Additional File 1, Table S2). Neither directional nor balancing selection was detected among microsatellites at any spatial scale within Manus Basin (LOSITAN, *P *> 0.05), but one microsatellite locus (*Ifr043*) was under positive selection (LOSITAN, 25,000 simulations, *P *< 0.001) at the basin level.

### Microsatellite marker identity and excluded markers

Identity tests were used to assess the utility of each microsatellite marker set. Within Manus Basin, probability of identity tests (*P_ID_*) and probability of sibling identity tests (*P_SIB_*) indicated that the nine microsatellite markers identify individuals (*P_ID _*= 1.5 × 10^-6^), including those that shared 50% genetic similarity (*P_SIB _*= 3.5 × 10^-3^). IMa coalescent models require that microsatellite markers adhere to the stepwise mutation model; only four of nine microsatellite markers (*Ifr043, Ifr052, Ifr078*, and *Ifr086*) adhered to this model and could be used for IMa analysis. Identity tests for these four markers suggested that they are insufficient for assessment of population structure (*P_ID _*= 1.1 × 10^-2^, *P_SIB _*= 0.12).

Within North Fiji and Lau Basins, four of nine microsatellite markers (*Ifr043, Ifr068, Ifr086*, and *Ifr103*) failed to amplify, were out of equilibrium, or were under selection. These markers were excluded from all analyses involving North Fiji and Lau Basins. The five remaining microsatellite loci could identify individuals (*P_ID _*= 4.0 × 10^-4^), even those that share 50% genetic similarity (*P_SIB _*= 4.4 × 10^-2^). Only two of those microsatellites (*Ifr052 *and *Ifr078*) adhered to the stepwise mutation model and could be used in IMa analyses. Identity tests for these two markers suggested that they are insufficient for assessment of population structure (*P_ID _*= 0.13, *P_SIB _*= 0.35).

### Population structure within Manus Basin

**Figure 2:**
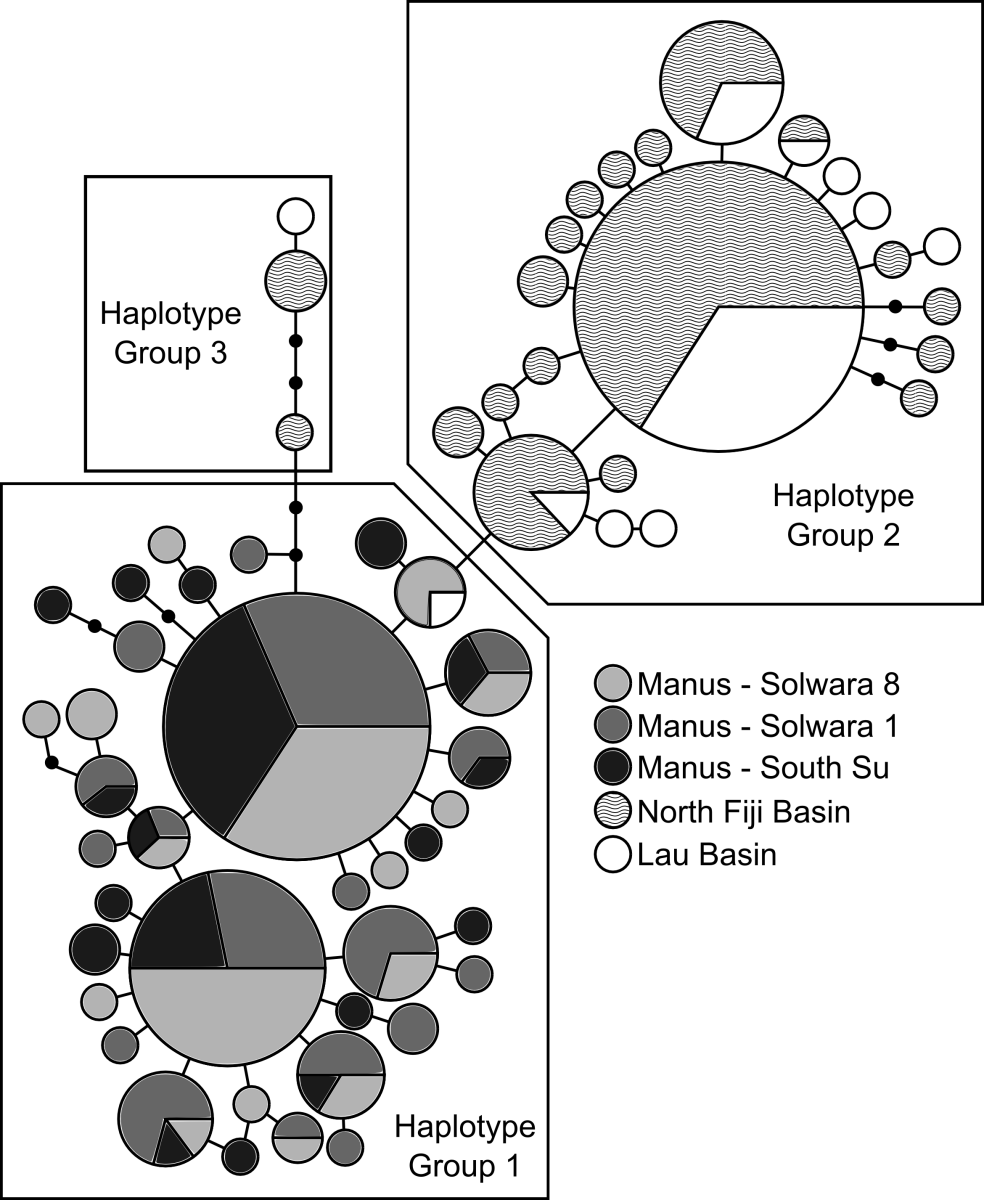
**Statistical parsimony network of *Ifremeria nautilei *haplotypes from Manus, North Fiji, and Lau Basin**. Area of circles is proportional to number of individuals that possess each haplotype. Small black circles represent inferred haplotypes not recovered in this data set. Each node represents a one base pair difference between haplotypes. Boxes delineate each putative haplotype group.

Mitochondrial genealogies revealed two frequent haplotypes at all Manus Basin sites (individuals per haplotype > 30; Figure 2). Less abundant haplotypes radiated from the dominant haplotypes in a star-like pattern (many shallow branches radiating from numerically dominant haplotypes). South Su contained the most private haplotypes (*n *= 10), followed by Solwara 1 (*n *= 8) and Solwara 8 (*n *= 7).

**Figure 3:**
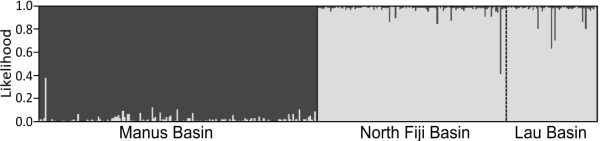
**Structure inferred two populations of *Ifremeria nautilei*, finding North Fiji and Lau Basin as one population distinct from Manus Basin**. Structure inferred two populations of *Ifremeria nautilei*, finding North Fiji and Lau Basin as one population distinct from Manus Basin. Manus Basin population indicated in black, North Fiji/Lau population indicated in grey. Solid black line denotes division between samples from Manus and North Fiji Basin. Dashed line indicates division between samples from North Fiji and Lau Basin. Eight microsatellite markers were used to test K (Additional File 1, Table S2). Likelihood plateaued at K = 2.

No indication of population structure was detected at the patch, mound, or site level within Manus Basin: Analysis of molecular variance (AMOVA) indicated no variation among haplotypes using either mitochondrial or microsatellite markers (*P *> 0.05); pairwise comparisons of *F_ST _*and *φ_ST _*revealed no significant genetic differentiation (*P *> 0.05); hierarchical analysis of *F_ST _*and *φ_ST _*did not reveal any significant population differentiation among nested samples (HIERFSTAT, *P *> 0.05; Table 3); assignment tests indicated that all *I. nautilei *collected in Manus Basin constitute a single population (Structure, *K *= 1, Figure 3).

**Table 3:** Hierarchical analysis of *F*-statistics from populations of *Ifremeria nautilei *sampled within Manus Basin using 9 microsatellite loci and a 404-bp region of the *COI *gene sequence and sampled among Manus, North Fiji, and Lau Basins using 5 microsatellite loci and a 404-bp region of the *COI *gene sequence.

Hierachical Level	9 Loci	COI
Patch/Mound	0.03 (0.27)	0.01 (0.17)
Mound/Site	0.01 (0.77)	0.01 (0.25)
Site/Total	0.00 (0.07)	0.01 (0.08)

Hierachical Level	5 Loci	COI

Site/Basin	0.00 (0.56)	0.01 (0.06)
Basin/Total	**0.05 (0.04)**	**0.23 (0.03)**

P-values indicated in parentheses.

### Population structure among Manus, North Fiji, and Lau Basin

Haplotypes present in Manus, North Fiji, and Lau Basins segregated into three groups (Figure 2). Haplotype group 1 included all samples from Manus Basin while haplotype group 2 contained a mix of individuals from North Fiji and Lau Basin (Figure 2). Haplotype group 3 also contained a mix of individuals from North Fiji and Lau Basin but was not directly connected to haplotype group 2 (Figure 2).

Assignment tests of individual multi-locus genotypes identified two geographical regions hosting distinct populations of *Ifremeria nautilei *(STRUCTURE, *K *= 2; Figure 3): one in Manus Basin and a second occupying North Fiji and Lau Basins. STRUCTURE output suggests that one individual from North Fiji Basin may be second-generation migrant from Manus Basin and one individual from Manus Basin may be a second-generation migrant from the North Fiji/Lau Basin population. Hierarchical analyses of *F*-statistics also detected significant differentiation at this regional level (HIERFSTAT, *P *< 0.05; Table 3), but no significant differentiation was detected at lower levels [among sites within regions, among sites within the Manus Basin, among mounds within Manus sites, or among patches within Manus mounds (Table 3)]. Pairwise *F_ST _*and *φ_ST _*values did not show a significant increase in differentiation with geographical distance among samples separated by less than 1100 km (Table 4).

**Table 4:** Pairwise comparison of *Ifremeria nautilei *populations from Manus, North Fiji, and Lau Basins.

	MB	NFB	LB
Manus Basin	-	**0.053**	**0.055**
North Fiji Basin	**0.514**	-	0.000
Lau Basin	**0.530**	-0.005	-

Pairwise F_ST _from 5 microsatellite loci reported above the diagonal, pairwise φ_ST _from 404-bp *COI *sequences reported below the diagonal. Significant differentiation after correction for multiple tests (*P *< 0.05) indicated in bold.

### Estimates of migration, effective population size, and divergence time

As reported above, the few microsatellites that adhered to the stepwise mutation model were insufficient to adequately assess gene flow. Only the mitochondrial *COI *data yielded consistent results in the IMa runs. Coalescent estimates of migration rate (IMa) could not rule out the possibility of equal and bidirectional migration within Manus Basin populations. Due to the high level of connectivity between Manus Basin samples, it is unlikely that estimates of gene flow would converge on the most likely solution. The posterior probabilities display multiple peaks and gradually increasing probabilities and thus results from these analyses should be approached with caution.

Across samples of *I. nautilei *from all three western Pacific basins, coalescent estimates of migration rate (IMa) suggest that migration between North Fiji and Lau Basin is high (Table 5). Consistent with a single panmictic population, *m*-values could not be constrained between North Fiji and Lau Basin (Additional File 2, Figure S1). No evidence for migration between Manus and either North Fiji or Lau Basin was detected (IMa), suggesting that *I. nautilei *from Manus Basin are isolated from North Fiji and Lau Basin (*m1 *= *m2 *= 0; Table 5). Posterior probability estimates for gene flow between North Fiji and Lau Basin were inconsistent, due to the fact that *Ifremeria nautilei *from North Fiji and Lau Basin are part of a single, undifferentiated population. An interpretation of directional migration between these two basins should be approached with caution.

**Table 5:** Best fit models for coalescent analysis of *Ifremeria nautilei *populations among Manus, North Fiji, and Lau Basin.

Population 1	Population 2	*θ _1_*	*θ _2_*	*θ _a_*	*m1*	*m2*	*τ*	/τ μ 1 *404	*τ/μ *2*404
Manus	North Fiji	59.3	23.3	0.1	0.0	0.0	2.09	103,000	345,700
Manus	Lau	66.0	12.6	0.4	0.0	0.0	1.55	79,900	256,400
Lau	North Fiji	66.9	66.9	6.7	6.2	27.2	0.25	12,400	41,400

*θ_1 _*= effective size of population 2, *θ_2 _*= effective size of population 2, *θ_a _*= effective size of ancestral population, *m_1 _*= migration into population 1 from population 2, *m_2 _*= migration in to population 2 from population 1, *τ *= splitting time between populations. Splitting time is calibrated against two mutations rates: μ_1 _= 5 × 10^-8 ^[21], μ_2 _= 1.5 × 10^-8 ^[76] adjusted for the number of base pairs in the sequence. Posterior probability densities are provided in Additional File 2, Figure S1.

For Manus, North Fiji, and Lau Basin samples of *Ifremeria nautilei*, estimates of effective population size using either microsatellite linkage disequilibrium or coalescent analysis could not constrain population sizes among basins, suggesting that within each basin, effective population size is functionally infinite. Estimates of splitting time place the oldest divergence between North Fiji and Manus Basin, with a relatively recent split between North Fiji and Lau Basin (Table 5).

## Discussion

The complete absence of genetic subdivision in populations of *Ifremeria nautilei *at distances up to 1000 kilometres suggests this species is able to colonize distant vent habitats and that the ciliated Warén's larvae produced by *I. nautilei *are adapted for long-distance dispersal, as hypothesized by Reynolds *et al*. [37]. Despite this dispersal potential, a barrier to gene flow exists between Manus and North Fiji/Lau Basin populations that are separated by 2500 kilometres. Although mitochondrial *COI *gene sequences and nuclear microsatellite loci are informative at different temporal scales, both markers indicated identical patterns of population structure in *I. nautilei*, regardless of spatial scale.

### Local population structure within Manus Basin

*Ifremeria nautilei *from the three Manus Basin sites belong to a single, panmictic population based on mitochondrial *COI *gene sequences and nuclear microsatellite markers. Although patch sizes were generally small, several patches (particularly in Solwara 1) had a sufficient sample size to test and reject the hypothesis that self-recruiting patches of *I. nautilei *might create the appearance of panmixia within this sample set.

*Ifremeria nautilei *from South Su had the highest abundance of private haplotypes and private alleles. Under a scenario of colonization with subsequent migration, this pattern could suggest that South Su might serve as a source population that contributes individuals to other sites sampled in Manus Basin. This directional gene flow is consistent with the path of the St. George's Undercurrent, which enters Manus Basin from the southeast and travels northwest, encountering South Su first, then Solwara 1 and Solwara 8, before merging with the Vitiaz Straight Undercurrent to form the New Guinea Coastal Undercurrent [43]. This hypothesis could be tested with development of additional genetic markers that provide more detailed genealogical information than the microsatellite markers used in this study and by sampling and analysis of individuals from sites further west that are known to support *Ifremeria nautilei*. Rapid population expansion, as suggested by Fu's *F_S _*and the star-like mitochondrial genealogies, could account for the emergence of private haplotypes and alleles at each sampled site within Manus Basin. Alternatively, the negative Fu's *F_S _*values could be a result of a selective sweep on the mitochondrial genome. Additional sequenced-based nuclear markers would be needed to rule out this possibility.

### Basin-scale population structure

Individuals of *Ifremeria nautilei *sampled from across the known range of the species in the south western Pacific could be subdivided genetically into two populations, one restricted to Manus Basin and one distributed throughout North Fiji and Lau Basins. Under an isolation-by-distance scenario, genetic differentiation is expected to gradually increase with distance [27], but distance alone does not appear to create a significant barrier to gene flow. North Fiji and Lau Basin samples, separated by ~1000 kilometres, were undifferentiated. Coalescent estimates of gene flow suggest that genetic isolation of *I. nautilei *populations between Manus Basin and North Fiji/Lau Basins may have existed, with occasional migration, for several hundred thousand generations (Table 5), but without an understanding of mutation rates and average generation times in these snails, it is impossible to place these estimates in a geological time frame.

The phylogeographic break between populations from Manus and North Fiji/Lau *Ifremeria nautilei *is striking, considering the high degree of mixing within each population. Phylogeographic breaks are often associated with oceanographic features (e.g., geomorphology, hydrology) that form effective dispersal barriers for a wide range of taxa (e.g., Cape Hatteras [44], Cape Cod [45], Easter Microplate [14]). A related provannid snail complex, *Alviniconcha *spp., occurs throughout southwestern Pacific basins [30] and is comprised of several cryptic species [35]. The observed phylogeographic isolation of *I. nautilei *populations thus does not reflect a pattern that is shared by other southwestern Pacific vent taxa.

The phylogeographic break between Manus and North Fiji/Lau populations of *Ifremeria nautilei *is not likely the result of a colonization event. Colonization would result in a founder effect, where the founded population contains a subset of alleles from the source population [46]. The single mitochondrial haplotype shared between the Manus and North Fiji/Lau populations is intermediate between dominant haplotypes from the two populations and may be a product of incomplete lineage sorting between formerly connected populations [47]. Haplotype Group 3 (North Fiji/Lau) is more closely related to Haplotype Group 1 (Manus Basin; Figure 2) and it consists of as many missing haplotypes as it does actual haplotypes. This abundance of missing haplotypes, relative to other haplotype groups, could be the result of inadequate sampling, or it may suggest that disproportionately more haplotypes in that lineage have gone extinct. Our interpretation is that the two populations once existed as a single population spanning Manus, North Fiji, and Lau Basins and that this population became isolated through a vicariant process that remains to be determined. The presence of potential second-generation immigrants in each population suggests that isolation might not be complete between the two regions..

While *Ifremeria nautilei *occurs throughout Manus, North Fiji, and Lau Basins, populations of *I. nautilei *follow the trend of greater endemism and limited connectivity hypothesized for species endemic to back-arc basin spreading centres [32]. In this context, it is not surprising that a population of *I. nautilei *is distributed through North Fiji and Lau Basin, as these two basins share genera and species [30]. Water masses tend to be retained within Lau Basin, with some movement of northwestward flowing undercurrents from Lau into North Fiji Basin (Thurnherr, unpublished data, available at http://www.ldeo.columbia.edu/~ant/LAUB-FLEX/). This undercurrent movement is consistent with the weak signal of directional gene flow from Lau into North Fiji Basin (Table 5). Likewise, the barrier to dispersal between North Fiji and Manus Basin that limits gene flow between *I. nautilei *populations is consistent with a barrier that was hypothesized to restrict species dispersal between these two basins [48]. The barrier could be caused by geomorphological obstacles (*i.e*. the Vanuatu Archipelago), the lack of depth overlap between sites in Manus Basin and sites in North Fiji and Lau Basin, or by a yet to be determined oceanographic feature.

## Conclusions

Theoretical and experimental studies suggest that spatially and temporally unstable environments favor broad dispersal capabilities [49,50], which in turn should lead to shallow or absent population subdivisions. In dynamic systems such as hydrothermal vents, where habitat availability is unpredictable, survival depends on long-distance dispersal of propagules. No significant genetic differentiation was found among samples of western Pacific *Ifremeria nautilei *at the patch, mound, or site levels within the Manus Basin. No differentiation was observed between samples of *I. nautilei *collected from North Fiji and Lau Basins, which are separated by ~1000 kilometres. The Manus Basin population of *I. nautilei *is isolated from that of the North Fiji and Lau Basins by an unknown process that limits contemporary gene flow.

Reproductive mode and larval type are often poor predictors of population structure in marine environments [3,40,41]. Species with broad dispersal potential have been reported with high levels of differentiation at spatial scales of a few kilometres or less [51-53], while species that would otherwise be expected to show fine-scale population structure have been reported to show surprisingly high levels of connectivity throughout their geographic range [54,55]. The absence of *Ifremeria nautilei *population structure at all but the broadest spatial scales is consistent with long-distance dispersal and the barrier to gene flow between the North Fiji and Lau Basin population and the Manus Basin population is likely extrinsic and not related to life history characteristics.

Fine-scale spatial sampling and genetic analysis such as that used in this study can inform mitigation and best-management practices for mineral extraction at deep-sea hydrothermal vents. The Solwara 1 site is targeted for deep-sea mineral extraction [56]. A robust understanding of population genetic structure at multiple spatial scales can define natural conservation units that can be used to minimize loss of genetic diversity within and among populations of vent-restricted species [57]. For *Ifremeria nautilei*, high rates of gene flow among the sampled Manus Basin sites suggests that the Solwara 1 vents are likely to be repopulated from other Manus Basin localities, including South Su and Solwara 8. Monitoring of species recovery and genetic diversity as the Solwara 1 population recovers after extraction operations cease should add insight into the rates at which novel haplotypes and alleles accumulate in this species, providing a means to estimate the ages and sizes of extant populations. The Manus Basin population of *I. nautilei *comprises a genetically distinct unit that should be managed separately from the North Fiji/Lau population.

## Methods

### Geographic setting, sample collection, and DNA extraction

*Ifremeria nautilei *were collected from three hydrothermal vent sites in Manus Basin: Solwara 8, Solwara 1, and South Su (Figure 1). One to four patches of *I. nautilei *were sampled from three mounds at each site (Table 1). Samples were collected during June-July 2008 with an ST212 trenching ROV modified for biological sampling. Foot tissue was preserved in 95% ethanol. Additional *I. nautilei *samples were acquired from a cruise that occurred during May-June 2005 with ROV *Jason II *from North Fiji and Lau Basins (Figure 1). Foot tissue was stored briefly at -20°C and transferred to 95% ethanol prior to DNA extraction. Genomic DNA was isolated by Chelex-Proteinase-K extraction as described in Thaler *et al*. [39] and extracted DNA was stored at 4°C until amplification.

### *COI *amplification and analysis

The mitochondrial *COI *(404-bp segment) region was amplified with the species-specific primers *COI*-3 and *COI*-6 [38] as follows: 10-100 ng DNA template, 10 × PCR Buffer (20 mM Tris, pH 8.8; 50 mM KCl; 0.01% Triton X-100; 0.02 mg/ml BSA), 2 mM MgCl_2_, 0.2 mM dNTP's, 0.5 μM each primer, and 1 unit Taq polymerase (Bioline: Taunton, MA) in a 20 μl final volume. Reaction conditions were as follows: 94°C for 1 minute; 30 cycles of 92°C for 40 s, 50°C for 60 s, 72°C for 90 s; final extension of 72°C for 5 min. Amplicons were verified on 1.8% agarose gels. To remove unincorporated nucleotides, 14 μl of PCR product was incubated with 0.2 μl 10 × ExoAP buffer (50 mM Bis-Tris, 1 mM MgCl_2_, 0.1 mM ZnSO_4_), 0.05 μl Antarctic Phosphatase (New England Biolabs: Ipswich, MA), 0.05 μl Exonuclease I (New England Biolabs: Ipswich, MA) at 37°C for 60 min followed by 85°C for 15 min to inactivate the enzymes. Bi-directional sequencing reactions were performed using the manufacturer's protocol for Big Dye Terminator Reaction (Applied Biosystems: Foster City, CA). Sequenced PCR product was purified using AMPure magnetic bead system (Agencourt: Morrisville, NC) following manufacturer's protocol, analyzed on an ABI 3730xl DNA Analyzer (Applied Biosystems International), and edited with Sequencher version 4.7 (Gene Codes: Ann Arbor, MI). Consensus sequences were compared against the NCBI GenBank database to confirm species identity [58] and aligned using the MUSCLE alignment algorithm [59]. A sequence for each unique haplotype was deposited in GenBank (North Fiji and Lau haplotypes - accession # JQ074110 to JQ074134; Manus Basin haplotypes - accession # JQ074135 to JQ074170).

Neighbor-joining phylograms of aligned mitochondrial sequences were assembled in MEGA version 4 [60] with an *Alviniconcha *sp. 2 as an outgroup. Statistical-parsimony networks were assembled in TCS version 1.21 (default settings; [61]). Arlequin version 3.11, [62] was used to estimate haplotype (*H*), nucleotide diversity (π), Fu's *F_s_*, and pairwise φ_ST_.

### Microsatellite methods

Nine microsatellite markers (*Ifr040, Ifr043, Ifr052, Ifr068, Ifr078, Ifr086, Ifr093, Ifr094*, and *Ifr103*) were amplified from Manus, North Fiji, and Lau Basin samples following methods reported in Thaler *et al*. [39]. To assess marker quality, allelic richness and divergence from Hardy-Weinberg Equilibrium (HWE) were calculated in GENEPOP (version 4.0, [63]). Permutation tests to determine significant variation in allelic richness were conducted in F-stat (version 2.9.3.2; [64]). Departures from HWE toward heterozygote excess or deficiency were assessed for each locus using GENEPOP exact tests. Loci were screened using LOSITAN to test for the potential influence of selection (25,000 simulations; [65]). Microsatellite markers that showed deviations from HWE expectations or found to be under the influence of selection were excluded from subsequent analyses. Identity tests (P_ID _and P_SIB_) were used to indicate whether a given set of microsatellites contains sufficient information to be useful for assessing population structure [66,67]. P_ID _and P_SIB _were calculated for all useful sets of microsatellite markers (Gimlet version 1.3.3; [68]).

### Common statistical methods

Arlequin version 3.11 was used to conduct analysis of molecular variance (AMOVA). HIERFSTAT [69,70] was used to assess hierarchical φ_ST _and F_ST _at various nested scales from patch to basin. Microsatellite Analyser (MSA; [71]) was used to identify significant differentiation between patches, mounds, sites, and basins. Alpha levels were adjusts via Sequential Bonferroni correction to account for multiple tests [72]. Structure version 2.3.3 [73] was used to visualize population structure. We used an admixture model with no *a priori *sample data and with sampling locations as prior distributions. Analyses were conducted with a 100,000 step burn-in, 1,000,000 Markov chain Monte Carlo repetitions, and 3 replicates per level from *K *= 1 to 12. The most likely *K *was identified by the average maximal value of *Ln P(D) *returned by Structure. The program LDNe [74] was used in an attempt to estimate effective population size based on linkage-disequilibrium between microsatellite loci.

### Isolation with migration

Migration rate (*m*), effective population size (θ), and divergence time (τ) between populations were estimated using the coalescent-based isolation-with-migration model implemented in IMa [75]. All estimates were scaled on mutation rates (μ) that are unknown for *COI *in *I. nautilei*, so splitting time was calibrated against two hypothetical rates: μ_1 _= 5 × 10^-8 ^(determined theoretically, see [21]), and μ_2 _= 1.5 × 10^-8 ^(borrowed from rates in the gastropod, *Littorina littorea*, see [76]). IMa runs were performed on *COI *and microsatellite data among three sites within Manus Basin and across all three basins. Only microsatellites that did not deviate from expectations for the stepwise mutation model could be used for IMa analyses [77].

A series of short (< 2,000,000 steps) IMa runs was conducted to optimize model parameters and determine the efficient priors for full runs. Prior probabilities and heating schemes were established at θ = 100, *m *= 100, and τ = 1.5, in a 40 chain geometric model. Effective sample size, autocorrelations, and trend plots were monitored to evaluate convergence. Three independent runs were compared to ensure that marginal posterior distributions had achieved similar solutions and results were averaged across the three runs. Generated trees were analysed in L-mode for best fit (default settings) and the most likely model was determined using Akaike Information Criterion [78] and 2LRR tests.

## Authors' contributions

CLVD, JC, TFS, and ADT conceived the study; ADT, TFS and RCV collected samples; ADT, KZ, WS, JC, and TFS undertook molecular benchwork and analyses; ADT, CC, RCV, KZ, and CLVD drafted the manuscript. All authors have read and approved the final manuscript.

## Supplementary Material

Additional file 1 **Table S1 - Summary statistics for nine microsatellite loci amplified from populations of Ifremeria nautilei within Manus Basin**. *n *= number of individuals, *a *= number of alleles, *Rs *= allelic richness, *P_A _*= number of private alleles, *HE *= expected heterozygosity *HO *= observed heterozygosity (bold = significant deviation from HWE, * = significant heterozygote excess, † = significant heterozygote deficiency, - = null amplification or monomorphic genotype). Patches that contained only 1 allele not shown. Table S2 - Summary statistics for eight microsatellite loci amplified from populations of Ifremeria nautilei from Manus, North Fiji, and Lau Basin. *n *= number of individuals, *a *= number of alleles, *Rs *= allelic richness, *P_A _*= number of private alleles, *HE *= expected heterozygosity *HO *= observed heterozygosity (bold = significant deviation from HWE, * = significant heterozygote excess, † = significant heterozygote deficiency, - = null amplification or monomorphic genotype). Patches that contained only 1 allele not shown. Click here for file

Additional file 2 **Figure S1 - Posterior probability densities for migration of Ifremeria nautilei between basins in the western Pacific, based on mitochondrial COI gene region**. Click here for file
