# Supplementary material for: The spatial scale of genetic subdivision in populations of Ifremeria nautilei, a hydrothermal-vent gastropod from the southwest Pacific
Source: BMC Evol Biol. 2011 Dec 22;11:372. doi: 10.1186/1471-2148-11-372 (PMC3265507; doi:10.1186/1471-2148-11-372)
Supplement: Additional file 1 — Table S1 - Summary statistics for nine microsatellite loci amplified from populations of Ifremeria nautilei within Manus Basin. n = number of individuals, a = number of alleles, Rs = allelic richness, PA = number of private alleles, HE = expected heterozygosity HO = observed heterozygosity (bold = significant deviation from HWE, * = significant heterozygote excess, † = significant heterozygote deficiency, - = null amplification or monomorphic genotype). Patches that contained only 1 allele not shown. Table S2 - Summary statistics for eight microsatellite loci amplified from populations of Ifremeria nautilei from Manus, North Fiji, and Lau Basin. n = number of individuals, a = number of alleles, Rs = allelic richness, PA = number of private alleles, HE = expected heterozygosity HO = observed heterozygosity (bold = significant deviation from HWE, * = significant heterozygote excess, † = significant heterozygote deficiency, - = null amplification or monomorphic genotype). Patches that contained only 1 allele not shown. [file 1471-2148-11-372-S1.DOC]

**Table S1 - Summary statistics for nine microsatellite loci amplified from populations of *Ifremeria nautilei* within Manus Basin.**

*n* = number of individuals, *a* = number of alleles, *Rs* = allelic richness, *P_A_* = number of private alleles, *HE* = expected heterozygosity *HO* = observed heterozygosity (bold = significant deviation from HWE, * = significant heterozygote excess, † = significant heterozygote deficiency, - = null amplification or monomorphic genotype). Patches that contained only 1 allele not shown.

**Table S2 - Summary statistics for eight microsatellite loci amplified from populations of *Ifremeria nautilei* from Manus, North Fiji, and Lau Basin.**

*n* = number of individuals, *a* = number of alleles, *Rs* = allelic richness, *P_A_* = number of private alleles, *HE* = expected heterozygosity *HO* = observed heterozygosity (bold = significant deviation from HWE, * = significant heterozygote excess, † = significant heterozygote deficiency, - = null amplification or monomorphic genotype). Patches that contained only 1 allele not shown.
