# Supplementary figures and images for: The spatial scale of genetic subdivision in populations of Ifremeria nautilei, a hydrothermal-vent gastropod from the southwest Pacific
Source: BMC Evol Biol. 2011 Dec 22;11:372. doi: 10.1186/1471-2148-11-372 (PMC3265507; doi:10.1186/1471-2148-11-372)

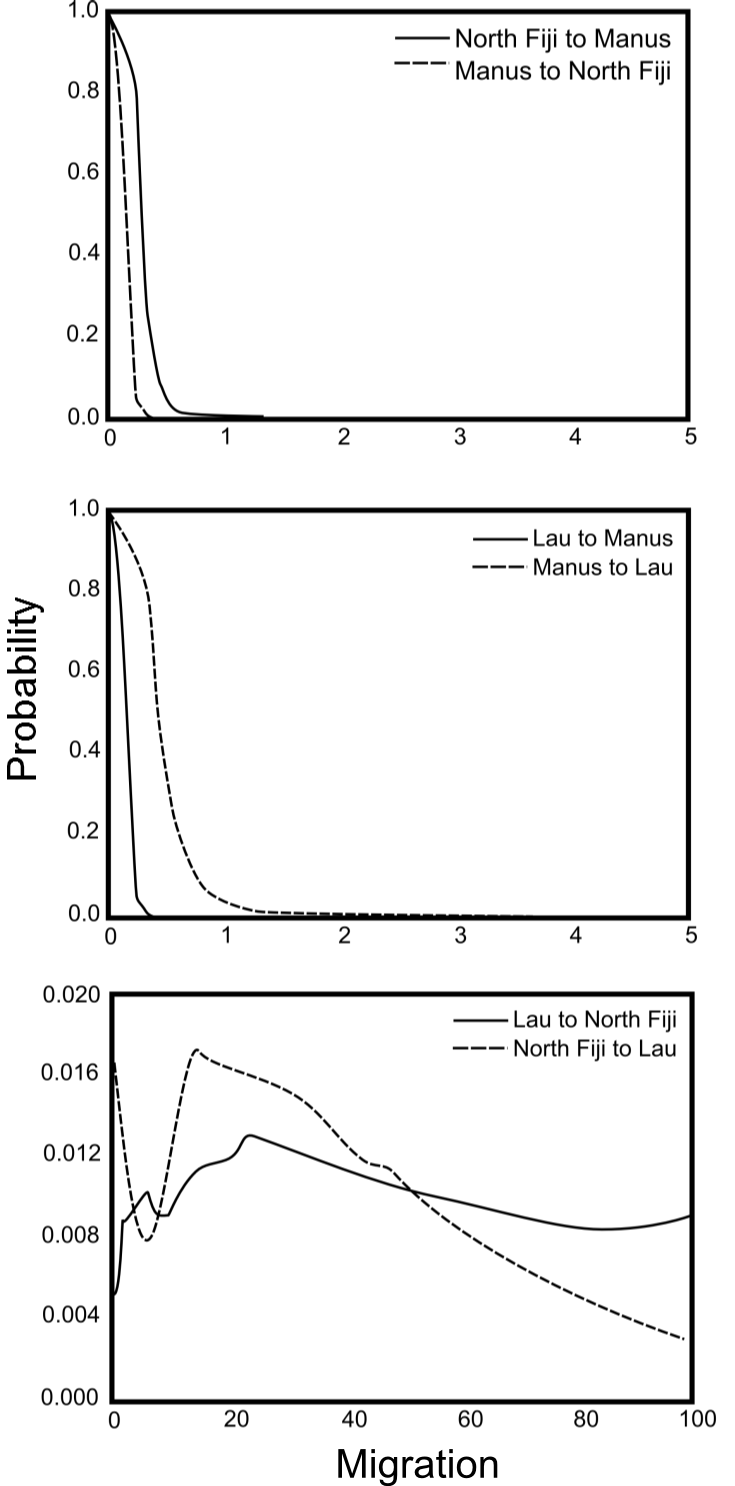

Supplement: Additional file 2 — Figure S1 - Posterior probability densities for migration of Ifremeria nautilei between basins in the western Pacific, based on mitochondrial COI gene region. [file 1471-2148-11-372-S2.PNG]
